# Threefold Increases in Population HIV Viral Load Suppression Among Men and Young Adults — Bukoba Municipal Council, Tanzania, 2014–2017

**DOI:** 10.15585/mmwr.mm6830a2

**Published:** 2019-08-02

**Authors:** Duncan MacKellar, Claire Steiner, Oscar E. Rwabiyago, Haddi J. Cham, Sherri Pals, Haruka Maruyama, Omari Msumi, Gerald Kundi, Johnita Byrd, Rachel Weber, Caitlin Madevu-Matson, Kokuhumbya Kazaura, Thomas Rutachunzibwa, Eunice Mmari, Fernando Morales, Jessica Justman, Kevin Cain, Anath Rwebembera

**Affiliations:** ^1^Division of Global HIV and TB, Center for Global Health, CDC; ^2^ICAP at Columbia University, Dar es Salaam, Tanzania; ^3^CDC Country Office, Dar es Salaam, Tanzania; ^4^CDC Country Office, Yaoundé, Cameroon; ^5^ICAP at Columbia University, New York City, New York; ^6^Ministry of Health, Community Development, Gender, Elderly and Children, Bukoba, Tanzania; ^7^National AIDS Control Program, Ministry of Health, Community Development, Gender, Elderly and Children, Dar es Salaam, Tanzania.

Reducing HIV-related morbidity and mortality, and effectively eliminating HIV transmission risk, depends on use of antiretroviral therapy (ART) to achieve and maintain viral load suppression (VLS)* (*1*,*2*). By 2020, sub-Saharan African countries are working to achieve VLS among 90% of persons using ART and 73% of all persons living with HIV infection (*1*). In Tanzania, a country with 1.4 million persons with HIV infection, 49.6% of HIV-positive persons aged 15–49 years had achieved VLS in 2017, including only 21.5% of men and 44.6% of women aged 25–29 years (*3*). To identify interventions that might increase VLS in Tanzania, and reduce VLS-associated sex and age-group disparities, the Bukoba Combination Prevention Evaluation (BCPE) scaled up new HIV testing, linkage to care, and retention on ART interventions throughout Bukoba Municipal Council (Bukoba), Tanzania, during October 2014–March 2017 (*4*,*5*). Located on the western shore of Lake Victoria, Bukoba is a mixed urban and rural municipality of 150,000 persons and capital of Kagera Region. Of the 31 regions of Tanzania, Kagera has the fourth highest prevalence of HIV infection (6.8%) among residents aged 15–49 years (*3*). CDC analyzed data from BCPE preintervention and postintervention surveys and found that VLS prevalence among HIV-positive Bukoba residents aged 18–49 years increased approximately twofold overall (from 28.6% to 64.8%) and among women (33.3% to 67.8%) and approximately threefold among men (20.5% to 59.1%) and young adults aged 18–29 years (15.6% to 56.7%). During 2017, BCPE facility–based testing and linkage interventions were approved as new service delivery models by the Tanzania Ministry of Health, Community Development, Gender, Elderly and Children (*4*,*5*). After a successful rollout to 208 facilities in 11 regions in 2018, BCPE interventions are being scaled up in all regions of Tanzania in 2019 with support from the United States President’s Emergency Plan for AIDS Relief (PEPFAR).^†^

BCPE interventions were implemented when national ART eligibility guidelines expanded from CD4 count <350/*μ*L (October 2014–November 2015) to ≤500/*μ*L (December 2015–September 2016) to any CD4 count (Test and Start^§^ [October 2016–March 2017]). HIV testing^¶^ was routinely offered at 11 participating health care facilities, in homes, and at community venues (Supplementary Figure, https://stacks.cdc.gov/view/cdc/80050) (*4*). Linkage case management** (services to help persons with HIV infection enroll early in HIV care) was offered to HIV-positive persons referred for care at participating facilities (*5*). Defaulter tracing^††^ (services to help patients resume HIV care among those who had stopped) was initiated for patients who defaulted from care during October 2014–March 2017 at nine participating facilities providing ART.

Household surveys conducted before (November 2013–January 2014) and after (June–September 2017) the interventions used identical methods to assess prior diagnosis of HIV infection, current ART use, and VLS among persons with HIV infection. In Bukoba census enumeration areas randomly selected in proportion to ward^§§^ population, all household members aged 18–49 years were eligible for an in-person interview and HIV testing. Specimens obtained from HIV-positive participants were tested at the national laboratory for HIV-1 viral load. Preintervention and postintervention prevalence, VLS prevalence ratios (PRs), and adjusted prevalence ratios (aPRs) were estimated using SAS (version 9.3; SAS Institute). All estimates were census-weighted by sex, age group, and geographic area, and adjusted for clustering within enumeration areas.

Among residents aged 18–49 years in sampled enumeration areas, 4,795 (73%) of 6,532 residents participated in preintervention survey interviews and HIV testing, and 5,067 (74%) of 6,844 residents participated in postintervention survey interviews and HIV testing. For both surveys, proportionally fewer men than women were contacted, interviewed, and tested for HIV infection (Figure).

**FIGURE:**
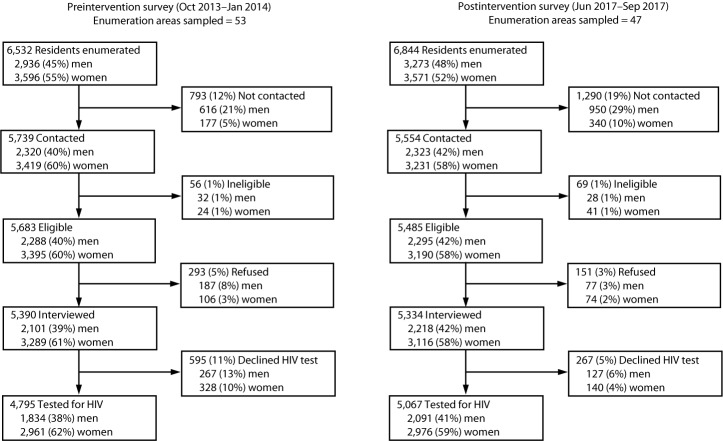
Participation in preintervention and postintervention household surveys to assess effectiveness of new HIV testing, linkage to care, and retention on antiretroviral therapy interventions — Bukoba Combination Prevention Evaluation, Bukoba Municipal Council, Tanzania, 2014–2017 **Abbreviation:** HIV = human immunodeficiency virus.

Before the intervention in 2014, among an estimated 66,134 residents aged 18–49 years, prevalence of HIV infection was 8.9%. Among the estimated 5,903 HIV-positive residents aged 18–49 years, 47.4% had previously received an HIV diagnosis, 40.8% were currently in HIV care, and 32.2% were using ART, 88.7% (95% confidence interval [CI] = 83.5–93.9) of whom had achieved VLS. Thus, an estimated 3,107 residents aged 18–49 years were unaware of their HIV infection and needed diagnosis, and 3,493 needed HIV care and ART if eligible, which served as intervention targets (Table 1).

**TABLE 1:** Preintervention prevalence of human immunodeficiency virus (HIV) infection among residents aged 18–49 years; preintervention prevalence of prior diagnosis of HIV infection, current enrollment in HIV care, and current use of antiretroviral therapy (ART) among HIV-positive residents aged 18–49 years; and intervention targets and outcomes — Bukoba Combination Prevention Evaluation, Bukoba Municipal Council, Tanzania, 2014–2017*

Characteristic	Preintervention prevalence estimates (Nov 2013–Jan 2014)^†^				Intervention targets and outcomes (Oct 2014–May 2017)^§^				
	HIV-positive	Prior HIV diagnosis	Currently in HIV care	Currently using ART	HIV-positive (total residents)	Need HIV diagnosis	HIV infection diagnosed^¶^	Need HIV care	Enrolled in HIV care^¶^
	% (95% CI)	% (95% CI)	% (95% CI)	% (95% CI)	No. (no.)	No. (%)**	No. (%)^††^	No. (%)**	No. (%)^§§^
**Total**	**8.9 (7.5–10.4)**	**47.4 (41.3–53.4)**	**40.8 (34.9–46.8)**	**32.2 (26.4–38.0)**	**5,903 (66,134)**	**3,107 (53)**	**3,381 (109)**	**3,493 (59)**	**3,488 (100)**
**Sex**									
Men	6.8 (5.2–8.4)	36.8 (28.0–45.5)	28.3 (20.0–36.6)	23.0 (14.9–31.1)	2,193 (32,435)	1,387 (63)	1,269 (91)	1,573 (72)	1,346 (86)
Women	11.0 (9.2–12.8)	53.6 (47.5–59.8)	48.3 (42.2–54.3)	37.7 (31.2–44.1)	3,710 (33,699)	1,720 (46)	2,112 (123)	1,920 (52)	2,142 (112)
**Age group (yrs)**									
18–24	3.9 (3.0–4.7)	28.8 (18.0–39.5)	23.2 (12.9–33.6)	15.4 (7.2–23.5)	856 (22,199)	609 (71)	809 (133)	657 (77)	737 (112)
25–49	11.5 (9.6–13.4)	50.5 (44.1–56.9)	43.8 (37.8–49.9)	35.1 (29.0–41.2)	5,047 (43,935)	2,498 (49)	2572 (103)	2,836 (56)	2,751 (97)

**Abbreviation:** CI = confidence interval.

* A community-wide combination prevention intervention was implemented throughout Bukoba Municipal Council during October 2014–March 2017 (Supplementary Figure, https://stacks.cdc.gov/view/cdc/80050). New interventions included comprehensive medical outpatient department and home- and venue-based HIV testing services; peer-delivered linkage case management for all consenting HIV-positive persons referred to 11 participating health care facilities; and defaulter-tracing services for patients at participating facilities who had not received HIV care in the prior 90 days during October 2014–March 2017. Recruitment into linkage case management ended March 30, 2017, and management services ended May 31, 2017.

^†^ The preintervention survey was conducted in census enumeration areas of Bukoba Municipal Council randomly selected in proportion to ward population in each of the 14 administrative wards. All household members aged 18–49 years were eligible to participate in a personal interview and test for HIV infection by the national rapid HIV test algorithm. Prevalence outcomes were estimated with SURVEYFREQ procedures, weighted by sex, age group, and geographic area of the Bukoba Municipal Council census and adjusted for clustering within census enumeration areas using SAS (version 9.3; SAS Institute). Prior diagnosis of HIV infection, and current enrollment in HIV care and use of ART (conditions) included persons with HIV infection who either 1) reported these conditions as part of the standard survey interview; 2) were confirmed by medical record to have these conditions; or 3) had achieved viral load suppression (<1,000 HIV RNA copies/*μ*L). Conditions included viral load suppression because of low sensitivity (77%) of self-reported ART use (https://www.ncbi.nlm.nih.gov/pubmed/29194115) and that only 1% of persons with HIV achieve durable viral load suppression in the absence of ART adherence (https://www.ncbi.nlm.nih.gov/pmc/articles/PMC3904947/). Percentages of conditions are of persons with HIV infection.

^§^ Bukoba Municipal Council census estimates were obtained from the Tanzania National Bureau of Statistics (https://www.nbs.go.tz/index.php/en/). Total residents = estimated census of residents aged 18–49 years. No. of HIV-positive residents = total residents multiplied by point HIV prevalence estimate. No. who need HIV diagnosis and no. who need HIV care = no. of HIV-positive residents minus (no. of HIV-positive residents multiplied by [point-prevalence estimates of prior HIV diagnosis and current enrollment in HIV care, respectively]). ART intervention targets were not established in 2014 because only HIV-positive persons with a CD4 count <350/*μ*L were eligible for ART.

^¶^ HIV diagnosed = clients aged 15–49 years who tested HIV-positive as part of combination prevention services (October 2014–March 2017), reported never previously testing positive for HIV infection, and were referred to Bukoba Municipal Council participating health care facilities. Enrolled in HIV care = linkage case management clients aged 15–49 years confirmed by medical record to have received HIV care at least once at participating health care facilities during October 2014–May 2017. Data on clients who received a new HIV diagnosis and enrolled in HIV care were collected by government-required age group intervals: 15–24 and 25–49 years. The percentage of clients aged 15–17 years is unknown; approximately 1% of ART patients at participating facilities are aged 15–17 years.

** Percentages are of estimated HIV-positive residents in 2014.

^††^ Percentages are of estimated HIV-positive residents in need of HIV diagnosis in 2014. Percentages >100% indicate that more HIV-positive persons aged 18–49 years received a new HIV diagnosis than expected based on census and preintervention survey point-prevalence estimates.

^§§^ Percentages are of estimated HIV-positive residents in need of HIV care in 2014. Percentages >100% indicate that more HIV-positive persons aged 18–49 years were enrolled in HIV care than expected based on census and preintervention survey point-prevalence estimates.

During the intervention (October 2014–March 2017), BCPE conducted 133,695 HIV tests; 4,731 clients of all ages tested positive for HIV and needed HIV care, 4,143 (88%) of whom received a new HIV diagnosis. Among 4,206 HIV-positive clients of all ages referred to participating facilities and who received BCPE linkage case management services, 3,918 (93%) enrolled in HIV care (3,186 before Test and Start), 2,521 (64%) of whom initiated ART within 3 months of diagnosis.

Among linkage case management clients who enrolled in care, an increasing proportion initiated ART within 3 months of diagnosis as national ART eligibility guidelines expanded: CD4<350 = 52% (1,057), CD4≤500 = 70% (815), and Test and Start = 89% (649). Of 820 patients who stopped HIV care and received BCPE defaulter-tracing services, 604 (74%) returned to care, and 573 (70%) initiated or reinitiated ART; an additional 830 patients were lost to follow-up (85% [706] before Test and Start). By the end of the intervention, BCPE achieved 109% and 100% of HIV diagnostic and enrollment-in-care targets for HIV-positive persons aged 18–49 years overall, and ≥91% and ≥86% for all sex and age groups, respectively (Table 1).

After the intervention in 2017, estimated prevalence of HIV infection among residents aged 18–49 years was 8.4% (95% CI = 6.9–9.9). Among HIV-positive residents aged 18–49 years, 76.2% (95% CI = 71.8–80.6) had previously received an HIV diagnosis, and 70.9% (95% CI = 65.6–76.3) were using ART, 91.3% (95% CI = 88.5–94.2) of whom had achieved VLS.

VLS prevalence among all persons with HIV infection increased approximately twofold overall (from 28.6% to 64.8%), among women (33.3% to 67.8%), and among those who had lived in their home for >2 years (34.4% to 70.4%). VLS prevalence increased approximately threefold among men (20.5% to 59.1%), persons aged 18–29 years (15.6% to 56.7%), and those who had unprotected sexual intercourse (17.7% to 54.9%) (Table 2). VLS sex and age group disparities in 2014 (aPR = 1.5–2.7) were nearly eliminated by 2017 (aPR = 1.1–1.3). With the exception of cell phone or television ownership, VLS prevalence disparities were not observed in 2017 for other sociodemographic characteristics (Table 2).

**TABLE 2:** Preintervention and postintervention household survey participant characteristics, population prevalence of viral load suppression (VLS)* among persons with human immunodeficiency virus (HIV) infection aged 18–49 years, and VLS prevalence ratios — Bukoba Combination Prevention Evaluation, Bukoba Municipal Council, Tanzania, 2014–2017^†^

Characteristic	Preintervention HIV survey^§^ (Nov 2013–Jan 2014)					Postintervention HIV survey^§^ (Jun 2017–Sep 2017)					Intersurvey VLS prevalence ratio^¶^
	Total	HIV-positive	VLS prevalence and prevalence ratios^¶^			Total	HIV-positive	VLS prevalence and prevalence ratios^¶^			
	No. (%)	No.	% (95% CI)	PR (95% CI)	aPR (95% CI)	No. (%)	No.	% (95% CI)	PR (95% CI)	aPR (95% CI)	PR (95% CI)
**Total**	**4,795 (100)**	**436**	**28.6 (23.0–34.2)**	**N/A**	**N/A**	**5,067 (100)**	**435**	**64.8 (59.4–70.2)**	**N/A**	**N/A**	**2.3 (1.8–2.8)**
**Sex**											
Men	1,834 (38.2)	113	20.5 (12.4–28.7)	Referent	Referent	2,091 (41.3)	112	59.1 (50.9–67.4)	Referent	Referent	2.9 (1.9–4.4)
Women	2,961 (61.8)	323	33.3 (27.2–39.5)	1.6 (1.1–2.4)	1.5 (1.1–2.1)	2,976 (58.7)	323	67.8 (61.8–73.8)	1.1 (1.0–1.3)**	1.1 (1.0–1.3)**	2.0 (1.7–2.5)
**Age group (yrs)**											
18–29	2,749 (57.3)	163	15.6 (8.6–22.5)	Referent	Referent	2,832 (55.9)	136	56.7 (47.9–65.4)	Referent	Referent	3.6 (2.3–5.7)
30–39	1,385 (28.9)	180	26.3 (20.0–32.7)	1.7 (1.1–2.7)	1.7 (1.1–2.8)	1,461 (28.8)	172	65.0 (57.9–72.2)	1.1 (1.0–1.3)**	1.2 (1.0–1.4)	2.5 (1.9–3.2)
40–49	661 (13.8)	93	52.5 (39.4–65.5)	3.4 (2.3–5.0)	2.7 (1.7–4.3)	774 (15.3)	127	72.8 (62.3–83.3)	1.3 (1.0–1.6)	1.3 (1.0–1.5)	1.4 (1.1–1.8)
**Duration of current home residence (yrs)**											
<1	1,559 (32.5)	137	19.9 (14.2–25.7)	Referent	Referent	1,519 (30.0)	142	57.1 (48.6–65.6)	Referent	Referent	2.9 (2.1–3.9)
1–2	1,059 (22.1)	99	28.0 (17.3–38.8)	1.4 (1.0–2.0)^**^	1.3 (0.9–1.9)	1,226 (24.2)	89	64.2 (54.0–74.4)	1.1 (0.9–1.4)	1.1 (0.9–1.3)	2.3 (1.5–3.4)
>2	2,177 (45.4)	200	34.4 (26.4–42.4)	1.7 (1.2–2.4)	1.3 (0.9–1.8)	2,322 (45.8)	204	70.4 (63.9–76.9)	1.2 (1.1–1.4)	1.1 (1.0–1.3)**	2.0 (1.6–2.6)
**Ownership of cell phone or television**											
No	364 (7.6)	68	22.4 (13.8–31.0)	Referent	Referent	209 (4.1)	44	46.7 (31.8–61.7)	Referent	Referent	2.1 (1.3–3.5)
Yes	4,431 (92.4)	368	29.8 (23.8–35.8)	1.3 (0.9–1.9)	1.2 (0.9–1.7)	4,858 (95.9)	391	67.2 (62.1–72.3)	1.4 (1.1–2.0)	1.4 (1.0–1.9)	2.3 (1.8–2.8)
**Trouble satisfying household food needs** ^††^											
Sometimes/Often/Always	250 (5.2)	46	25.7 (12.8–38.6)	Referent	—	637 (12.6)	108	67.8 (58.0–77.7)	Referent	—	2.6 (1.6–4.3)
Seldom	1,822 (38.1)	196	28.1 (21.0–35.1)	1.1 (0.6–1.9)	—	2,118 (42.0)	206	64.7 (57.8–71.7)	1.0 (0.8–1.1)	—	2.3 (1.7–3.0)
Never	2,707 (56.6)	193	29.9 (21.6–38.2)	1.2 (0.7–2.0)	—	2,285 (45.3)	120	62.0 (52.0–71.9)	0.9 (0.7–1.1)	—	2.1 (1.5–2.8)
**Highest level of education completed** ^††^											
None/Some primary	556 (11.6)	92	24.5 (15.6–33.4)	Referent	Referent	532 (10.5)	83	66.4 (56.6–76.1)	Referent	—	2.7 (1.8–4.0)
Completed primary	2,596 (54.2)	279	31.7 (24.2–39.1)	1.3 (0.9–1.9)	0.8 (0.5–1.3)	2,546 (50.3)	276	64.6 (58.4–70.8)	1.0 (0.8–1.1)	—	2.0 (1.6–2.6)
Post primary	1,640 (34.2)	65	21.3 (11.8–30.8)	0.9 (0.5–1.6)	1.1 (0.7–1.5)	1,987 (39.2)	76	63.6 (52.2–75.0)	1.0 (0.8–1.2)	—	3.0 (1.9–4.7)
**Sexual behavior in the past 6 mos** ^§§^											
Unprotected intercourse	3,019 (63.0)	234	17.7 (12.3–23.2)	Referent	Referent	3,380 (66.7)	192	54.9 (46.9–63.0)	Referent	Referent	3.1 (2.2–4.3)
Protected intercourse	1,122 (23.4)	106	41.3 (30.6–52.0)	2.3 (1.6–3.4)	1.9 (1.3–2.7)	1,067 (21.1)	151	73.7 (66.5–80.8)	1.3 (1.1–1.6)	1.3 (1.1–1.6)	1.8 (1.4–2.3)
No sexual partners	654 (13.6)	96	42.0 (29.3–54.6)	2.4 (1.7–3.4)	1.9 (1.3–2.7)	620 (12.2)	92	72.7 (60.4–84.9)	1.3 (1.1–1.6)	1.2 (1.0–1.5)	1.7 (1.2–2.4)

**Abbreviations:** aPR = adjusted prevalence ratio; CI = confidence interval; N/A = not applicable; PR = prevalence ratio.

* HIV-1 RNA concentration <1,000/*µ*L on a viral load assay.

^†^ A community-wide combination prevention intervention was implemented throughout Bukoba Municipal Council during October 2014–March 2017 (Supplementary Figure, https://stacks.cdc.gov/view/cdc/80050). New interventions included comprehensive medical outpatient department, and home- and venue-based HIV testing services; peer-delivered linkage case management for all consenting HIV-positive persons referred to 11 participating health care facilities; and defaulter-tracing services for patients at participating facilities who had not received HIV care in the prior 90 days during October 2014–March 2017.

^§^ Preintervention and postintervention household surveys were conducted with identical survey methods and instruments. In census enumeration areas of Bukoba Municipal Council randomly selected in proportion to each of the 14 administrative ward populations, all household members aged 18–49 years were eligible to participate in a personal interview and test for HIV infection by the national rapid HIV test algorithm. Specimens of HIV-positive participants were tested at the national laboratory for HIV-1 viral load.

^¶^ VLS prevalence among persons with HIV infection, VLS PRs, and VLS aPRs were estimated with SURVEYFREQ and GENMOD procedures, weighted by sex, age group, and geographic area of the Bukoba Municipal Council census, and adjusted for clustering within census enumeration areas using SAS (version 9.3, SAS Institute). Variables noted with a dash for aPR were not included in the multivariate GENMOD model. Unless otherwise indicated, all PRs with a lower bound of the 95% CI ≥1.0 are statistically significant (p<0.05).

** p≥0.05.

^††^ Subtotals do not sum to total participants or total HIV-positive because of missing responses.

^§§^ Unprotected = condom use for <100% of sexual intercourse acts; protected = condom use for 100% of sexual intercourse acts.

## Discussion

After implementation of a new community-wide combination prevention intervention in Bukoba during a 2.5-year period of expanding ART eligibility, VLS prevalence among HIV-positive residents aged 18–49 years increased approximately twofold overall and approximately threefold among men and young adults aged 18–29 years, two groups known to have low VLS coverage in Tanzania and elsewhere (*1*,*3*). Although benefiting from only 6 months of Test and Start (ART for all HIV-positive persons), BCPE nearly achieved the 73% VLS prevalence target for women, persons aged 40–49 years, and residents living in their current home >2 years. Findings from BCPE suggest that comprehensive medical outpatient department and community-based HIV testing strategies, combined with Test and Start and recommended linkage and defaulter-tracing services, can substantially increase VLS prevalence and reduce VLS-associated sex and age group disparities in a relatively short time.

Although not measured in BCPE, reduction in incidence of HIV infection in Bukoba since 2014 is possible based on the large increase in VLS prevalence, including a threefold increase in VLS among HIV-positive persons who had unprotected sexual intercourse (*2*). In a community-randomized trial in Botswana, annual incidence of HIV infection was reduced approximately 30% in intervention communities with a smaller net increase, but higher VLS prevalence, compared with BCPE (*6*).

A Test and Start trial conducted in 16 rural communities of approximately 5,000 residents each in Kenya and Uganda increased VLS prevalence among HIV-positive residents by an absolute difference of 35.5% (from 44.7% to 80.2%) (*7*). In a 2-year period, ART was provided to all persons with HIV infection through community health campaigns and home-based HIV testing services, an intervention strategy that might not be as effective in larger urban communities such as Bukoba (*7*). BCPE interventions met or exceeded overall targets for diagnosis of HIV infection and enrollment-in-care, helping to increase VLS prevalence by a similar absolute difference of 36.2% (from 28.6% to 64.8%). However, the 73% VLS prevalence target was not met, in part, because most (81%) BCPE HIV-positive clients were enrolled in care before Test and Start and many patients had defaulted from HIV care. Although comprehensive defaulter-tracing services included retracing defaulters, many patients were lost to follow-up (706 before Test and Start). Implemented under nonexperimental, real-world conditions, BCPE findings are consistent with reports of low retention in HIV care in sub-Saharan Africa before Test and Start (*8*). Thus, achieving ≥73% VLS prevalence among persons with HIV infection in Tanzania might not only depend on optimizing HIV testing and ART linkage services, but also on concerted efforts to improve retention and identify and return to ART care many patients who might have defaulted before Test and Start (*8*).

Notably, exceeding the preintervention target for testing HIV-positive persons in need of diagnosis (109%) should have resulted in a higher postintervention prevalence of prior diagnosis of HIV infection (76%). Beyond uncertainty of census and sample survey prevalence estimates, two reasons likely explain this difference. First, although intervention target counts of clients who received a new HIV diagnosis were restricted to those referred to Bukoba facilities, some of these clients might not have resided in Bukoba (which is home to the regional referral hospital and two health centers known to provide medical services to residents from other districts). Second, because comprehensive testing, linkage, and retention interventions were not scaled up in other districts, fewer persons who had previously received an HIV diagnosis might have moved into than out of Bukoba during the 2.5-year intervention (differential migration). Differential migration might have contributed to potentially lower VLS prevalence in 2017 among persons with HIV infection who reported living in their home for <1 year (57%) compared with >2 years (70%).

The findings in this report are subject to at least four limitations. First, because the evaluation did not include control communities, the effect of BCPE interventions on population VLS prevalence could not be estimated. Second, although prevalence estimates were weighted to the census population, residual bias might reduce the validity of estimates for men who were underrepresented in both surveys. Third, residence of clients who received BCPE testing and linkage services was not collected and is unknown. Finally, despite adjustment for VLS, estimated prevalence of prior diagnosis of HIV infection and ART use might be underestimated because of low sensitivity of self-report (*9*).

In 2017, BCPE facility-based HIV testing and linkage case management interventions were approved as new service delivery models by the Ministry of Health, and were implemented in 2018 by four nongovernmental organizations in 208 health care facilities and as part of community-based services in 11 regions of Tanzania (*4*,*5*). PEPFAR is supporting the nationwide scale-up of BCPE interventions in Tanzania in 2019 and recommends optimized provider-initiated HIV testing services and peer-delivered, linkage case management as potential strategies for countries to help achieve ≥73% prevalence of VLS among all persons with HIV infection by the end of 2020 (*10*).

**Summary**

**What is already known about this topic?**

Achieving and sustaining viral load suppression (VLS) reduces illness and death associated with human immunodeficiency virus (HIV) infection and effectively prevents sexual transmission of HIV.

**What is added by this report?**

In Bukoba, Tanzania, scale-up of new testing, linkage to care, and retention on antiretroviral therapy interventions over 2.5 years helped increase VLS among HIV-positive persons approximately twofold overall (from 28.6% to 64.8%) and threefold among men (20.5% to 59.1%) and adults aged 18–29 years (15.6% to 56.7%).

**What are the implications for public health practice?**

During 2019, these interventions are being scaled up across Tanzania with support from the U.S. President’s Emergency Plan for AIDS Relief to help increase VLS among all persons with HIV infection.

## Footnotes

* HIV-1 RNA concentration <1000/*μ*L on a viral load assay.

† https://www.pepfar.gov/documents/organization/285852.pdf.

§ Provision of ART for all persons living with HIV infection, regardless of CD4 count. https://www.pepfar.gov/documents/organization/250048.pdf.

¶ All patients seeking outpatient department medical services at three faith-based and all eight government health care facilities (excluding police and military) were routinely screened and offered HIV testing if eligible by national guidelines. HIV testing was offered at least once at all homes throughout Bukoba and at 79 venues frequented by men (e.g., businesses, bars, community events, and high-traffic urban areas). https://journals.plos.org/plosone/article?id=10.1371/journal.pone.0215654.

** Includes the following peer-delivered services recommended by CDC and the World Health Organization provided for ≤90 days: 1) point-of-diagnosis psychosocial support and counseling on the benefits of early enrollment in HIV care and ART; 2) escort and treatment navigation at HIV-care facilities; 3) periodic telephone calls and appointment reminders; and 4) follow-up in-person counseling on disclosure of HIV infection status, HIV testing of partners and family members, and identifying and resolving real and perceived barriers to HIV care. https://journals.plos.org/plosone/article?id=10.1371/journal.pone.0208919.

†† Includes telephone, home visit, escort, and treatment navigation services to help patients who had not received care in >90 days return to care and initiate or reinitiate ART if eligible. Electronic medical records were reviewed quarterly during July 2016–April 2017 to identify defaulters. Patients not contacted or contacted but who did not return to care were retraced in subsequent quarters.

§§ Bukoba Municipal District is composed of 14 administrative wards. (Supplementary Figure, https://stacks.cdc.gov/view/cdc/80050).

## References

[1] Joint United Nations Programme on HIV/AIDS. 90–90–90: an ambitious treatment target to help end the AIDS epidemic. Geneva, Switzerland: Joint United Nations Programme on HIV/AIDS, 2014. https://www.unaids.org/en/resources/documents/2014/90-90-90

[2] CDC. Evidence of HIV treatment and viral suppression in preventing the sexual transmission of HIV. Atlanta, GA: US Department of Health and Human Services, CDC; 2018. https://www.cdc.gov/hiv/risk/art/index.html

[3] Tanzania Commission for AIDS (TACAIDS), Zanzibar AIDS Commission (ZAC). Tanzania HIV Impact Survey (THIS) 2016–2017: final report. Dar es Salaam, Tanzania: United Republic of Tanzania Ministry of Health, Community Development, Gender, Elderly and Children; 2018. https://phia.icap.columbia.edu/countries/tanzania/

[4] Cham HJ, MacKellar D, Maruyama H, Methods, outcomes, and costs of a 2.5 year comprehensive facility-and community-based HIV testing intervention in Bukoba Municipal Council, Tanzania, 2014–2017. PLoS One 2019;14:e0215654. 10.1371/journal.pone.0215654 31048912 PMC6497243

[5] MacKellar D, Maruyama H, Rwabiyago OE, Implementing the package of CDC and WHO recommended linkage services: methods, outcomes, and costs of the Bukoba Tanzania Combination Prevention Evaluation peer-delivered, linkage case management program, 2014–2017. PLoS One 2018;13:e0208919. 10.1371/journal.pone.0208919 30543693 PMC6292635

[6] Makhema J, Wirth KE, Pretorius Holme M, Universal testing, expanded treatment, and incidence of HIV infection in Botswana. N Engl J Med 2019;381:230–42. 10.1056/NEJMoa1812281 31314967 PMC6800102

[7] Petersen M, Balzer L, Kwarsiima D, Association of implementation of a universal testing and treatment intervention with HIV diagnosis, receipt of antiretroviral therapy, and viral suppression in East Africa. JAMA 2017;317:2196–206. 10.1001/jama.2017.5705 28586888 PMC5734234

[8] Plazy M, Orne-Gliemann J, Dabis F, Dray-Spira R. Retention in care prior to antiretroviral treatment eligibility in sub-Saharan Africa: a systematic review of the literature. BMJ Open 2015;5:e006927. 10.1136/bmjopen-2014-006927 26109110 PMC4479994

[9] Grabowski MK, Reynolds SJ, Kagaayi J, The validity of self-reported antiretroviral use in persons living with HIV: a population-based study. AIDS 2018;32:363–9. 10.1097/QAD.0000000000001706 29194115 PMC6171354

[10] United States President’s Emergency Plan for AIDS Relief. PEPFAR solutions platform. Washington, DC: United States President’s Emergency Plan for AIDS Relief; 2018. https://www.pepfarsolutions.org/solutions

